# Integer Programming-Based Method for Designing Synthetic Metabolic Networks by Minimum Reaction Insertion in a Boolean Model

**DOI:** 10.1371/journal.pone.0092637

**Published:** 2014-03-20

**Authors:** Wei Lu, Takeyuki Tamura, Jiangning Song, Tatsuya Akutsu

**Affiliations:** 1 Bioinformatics Center, Institute for Chemical Research, Kyoto University, Gokasho, Uji, Kyoto, Japan; 2 Department of Biochemistry and Molecular Biology, Monash University, Melbourne, Australia; 3 National Engineering Laboratory for Industrial Enzymes, Tianjin Institute of Industrial Biotechnology, Chinese Academy of Sciences, Tianjin, China; University of Georgia, United States of America

## Abstract

In this paper, we consider the Minimum Reaction Insertion (MRI) problem for finding the minimum number of additional reactions from a reference metabolic network to a host metabolic network so that a target compound becomes producible in the revised host metabolic network in a Boolean model. Although a similar problem for larger networks is solvable in a flux balance analysis (FBA)-based model, the solution of the FBA-based model tends to include more reactions than that of the Boolean model. However, solving MRI using the Boolean model is computationally more expensive than using the FBA-based model since the Boolean model needs more integer variables. Therefore, in this study, to solve MRI for larger networks in the Boolean model, we have developed an efficient Integer Programming formalization method in which the number of integer variables is reduced by the notion of feedback vertex set and minimal valid assignment. As a result of computer experiments conducted using the data of metabolic networks of *E. coli* and reference networks downloaded from the Kyoto Encyclopedia of Genes and Genomes (KEGG) database, we have found that the developed method can appropriately solve MRI in the Boolean model and is applicable to large scale-networks for which an exhaustive search does not work. We have also compared the developed method with the existing connectivity-based methods and FBA-based methods, and show the difference between the solutions of our method and the existing methods. A theoretical analysis of MRI is also conducted, and the NP-completeness of MRI is proved in the Boolean model. Our developed software is available at “http://sunflower.kuicr.kyoto-u.ac.jp/~rogi/minRect/minRect.html.”

## Introduction

Metabolism is one of the most important biological processes in organisms. Relations between reactions and chemicals in the metabolism are often represented by metabolic networks [1]. Since many of these metabolic processes can produce commodity and specialty chemicals, the manipulation of metabolisms has been extensively studied in the field of metabolic engineering. One of the most successful applications of metabolic engineering is production of industrially valuable products using a microbial host with recombinant technologies [2]–[4]. Techniques for production of desired chemicals using a microbial host are roughly classified into the following three types [5]: (a)combinations of existing pathways, (b)engineering of existing pathways, and (c) *de novo* pathway design. In (a), partial pathways can be recruited from independent organisms and co-localized in a single host. For example, 1,3-propanediol is synthesized by Nakamura *et al.* in which pathways from *Saccharomyces cerevisiae* and *Klebsiella pneumonia* were assembled *in E. coli* [6] and another example is the production of artemisinic acid, a precursor to the plant-based anti-malarial drug artemisinin in yeast [7]. In (b), new non-natural chemicals can be produced by engineering existing routes [8], [9]. (c) is realized by the combination of (a) and (b), that is, the recruitment of partial pathways from different species and the use of engineered enzymes for extensions of pathways. It is to be noted that (a) focuses on the topology of the given metabolic networks, while (b) and (c) utilize the information of the structures of chemicals as well.

The “pathway prediction system” (PPS) of the University of Minnesota Biocatalysis and Biodegradation Database (UM-BBD) is designed to predict routes for the biodegradation of xenobiotic compounds [10]–[12]. From a set of previously defined biotransformation rules, the PPS guides the user through potential pathways one step at a time, requiring the selection of a new target metabolite at each step [5]. Biochemical Network Integrated Computational Explorer (BNICE) is a computational framework for generating every possible biochemical reaction from a given set of enzyme reaction rules and source or target compounds [13], [14]. However, since the number of predicted novel pathways is huge in many cases, some prioritization is necessary to choose the most promiscuous ones [15]. For example, one measure of such prioritization is to minimize the number of enzymatic steps [16].

In the type (a) problem, it seems that there are three major models for judging the producibility of target compounds, that is, *connectivity model*, *flow model*, and *Boolean model*. For each of them, Minimum Reaction Insertion (MRI) problem can be defined for finding the minimum number of additional reactions from a reference metabolic network to a host metabolic network so that a target compound becomes producible in the revised host metabolic network. In the connectivity model such as [16], the producibility of target compounds is judged by the connectivity between the source and the target compounds. After the source and the target compounds are connected by the additional reactions, the producibility is often evaluated by such a flow model as flux balance analysis (FBA) or an elementary mode [17], in which the sum of incoming flows must be equal to the sum of outgoing flows for each compound and the ratio of the amount of substrates and products must satisfy the coefficients given in each chemical reaction formula. In the Boolean model, each reaction occurs if all its substrates are producible whereas each compound is producible if one of its producing reactions occurs [18]. The source compounds are called *seeds* and the producible compounds are called the *scope* of the seed. In this model, a Boolean function of “AND” is attached to each reaction node and “OR” is attached to each compound node in the metabolic networks.

For example, suppose that there is a chemical reaction “A+B→C+D”, where A and B are called *substrates* whereas C and D are called *products*. In the connectivity model, either A or B is necessary to produce C and D, whereas both A and B are necessary for the Boolean model. In the flow model including FBA, in addition to the condition that both A and B must exist, both C and D are necessary to be consumed by other reactions. Thus, each model outputs a different solution for producing desired compounds.

From the view point of computational complexity, although the connectivity model is very simple and then applicable even to very large networks, its logical analysis ability is not strong since it cannot detect the lack of necessary substrates. The good point of the flow model is its computational efficiency since problems in the flow model can often be formalized by linear programming, for which there exist polynomial time algorithms [19]. However, these polynomial time algorithms are not applicable for MRI since discrete variables are necessary for representing additional reactions, although it is solvable by mixed integer programming [20].

Although the computational time of the FBA-based method for MRI is very small and scalable for genome-scale metabolic reconstruction [20], Boolean methods also have attractive features and are expected to complement the FBA-based method. Indeed, for the analysis of metabolic networks, many studies have been conducted to develop Boolean models. For example, Lemke *et al.* [21] studied the effect of deletion of each enzyme in the metabolic network of a Boolean model, and Smart *et al.* [22] considered almost the same problem from the viewpoint of the Boolean aspect of the flux balance model. Li *et al.* [23] and Sridhar *et al.* [24] have developed methods for finding a set of enzymes whose inhibition stops the production of the target compounds with a minimum elimination of the non-target compounds. Lee *et al.* [25] and Takemoto *et al.* [26] estimated the distribution of the size of the effect of the deletions of enzymes using a branching process.

**Figure 1:**
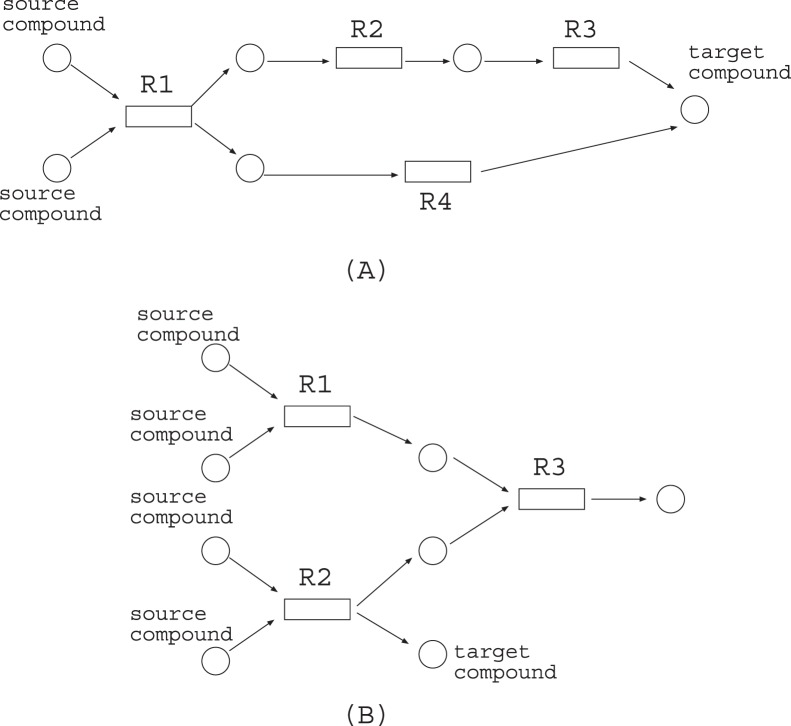
A problem of how to produce a target compound from the source nodes. In the Boolean model, either {R1, R4} or {R1, R2, R3} is sufficient, whereas {R1, R2, R3, R4, R5} is necessary for the flow model including FBA.

As for the shortcoming of the FBA-based method for MRI, it tends to be considerably affected by the redundancy of the given metabolic network since each node is affected not only by the incoming flows but also by the outgoing flows. For example, suppose that a metabolic network of Fig. 1 (A) is given, where circles and rectangles represent compounds and reactions respectively. In order to produce the target compound from the source compounds, {R1, R2, R3, R4} is necessary in the flow model including FBA, whereas either {R1, R4} or {R1, R2, R3} is sufficient for the Boolean model. Moreover, in the metabolic network of Fig. 1 (B), {R1,R2,R3} is necessary for FBA whereas {R2} is sufficient for the Boolean model.

Therefore, in this research, we study the problem of designing a pathway for producing target compounds in metabolic networks of the Boolean model since its logical analysis ability is more stable than that of the FBA, particularly when the flexible parts of the metabolic networks are large. Our approach is based on (a), that is, the combination of existing pathways. In our problem setting, a base metabolic network of a host organism, which we call the *host network*, is given; it cannot produce the target compound in its initial form. However, an integrated metabolic network of many other organisms are given as the *reference network* from which we should find the minimum number of additional reactions so that the target compound becomes producible. We prove that this problem is NP-complete.

Although both the FBA-based model and the Boolean model for MRI are considered to be NP-complete, the former is likely to have a faster exponential time algorithm than the latter since FBA has fewer integer variables. Although the computational complexity of the Boolean model is large, we develop an efficient method based on integer programming (IP) [27], [28], which is often used as a formalization of NP-complete problems and there is an efficient free solver for IP called CPLEX [29]. We also conducted four computer experiments in which the metabolic network of *E. coli* is used as the host network and the reference pathway of the KEGG database [30] is used as the reference network, and propanol, butanol, sedoheptulose 7-phosphate, and maleic acid are used as the target compound in each experiment. The results of the experiments show that (1) our IP-based method can appropriately solve MRI in the Boolean model; (2) solutions of MRI in the Boolean model are more suitable than those by connectivity based methods; (3) our IP-based method is applicable to large-scale networks where an exhaustive search does not work; and (4) solutions of MRI in the Boolean model tend to be smaller than those in the FBA-based model based on [31]. Our developed software is available at “http://sunflower.kuicr.kyoto-u.ac.jp/~rogi/minRect/minRect.html”.

## Materials and Methods

### Problem Definition

In this section, the main problem **Minimum Reaction Insertion (MRI)** in a Boolean model is first explained with an example and then mathematical formalization is described.

**Figure 2:**
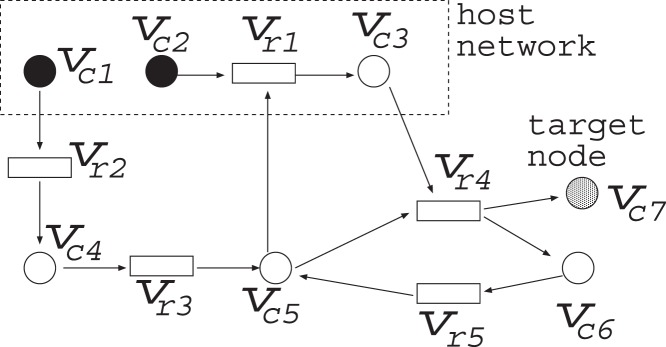
An example of MRI. 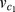
 and 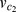 are the source nodes.

Suppose that a metabolic network shown in Fig. 2 is given, where each rectangle (resp., circle) corresponds to a reaction (resp., chemical compound). For example, 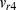 is a reaction, its substrates are 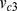 and 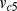 and its products are 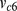 and 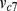. Black circles 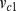 and 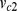 denote the source nodes and are assumed to be provided by the external environment. On the other hand, a gray circle 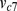 represents a target compound and the purpose of MRI is to make the target compound producible. However, initially only the host network, which is shown by the dotted rectangle, is available. Since only 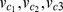 and 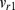 are included in the host network, the target compound 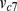 is not producible. Instead the entire network is called the reference network and reactions not included in the host network can be added later. In MRI, the minimum number of additional reactions should be determined to make the target compound producible. In this example, the addition of 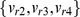 is the optimal solution. The difficult point of MRI is how to deal with the effect of cycles. In the example of Fig. 2, the addition of 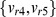 looks like the optimal solution. However, this solution is not appropriate since it relies on the cycle consisting of 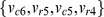 and 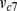 is not producible unless the initial amount of 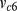 is sufficiently large.

MRI is mathematically defined as follows: A *metabolic network* can be represented by a directed graph 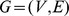. There are two types of node sets 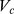 and 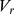, where 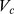 denotes a set of *compound nodes* and 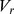 represents a set of *reaction nodes*. 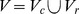 and 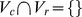 hold. The neighbors of compound nodes must be reaction nodes, and the neighbors of reaction nodes must be compound nodes. Let 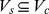 be a set of *source nodes* and 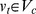 be a *target node*. Source nodes have no incoming edges and correspond to the seed compounds of [18]. In this study, we assume that source nodes are producible at any time.

Suppose that a *host network* 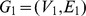 and a *reference network* 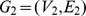 are given where 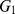 and 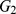 are metabolic networks, and 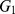 is a subgraph of 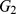 induced by 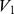. 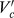 (resp., 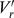) is a set of compound nodes (resp., reaction nodes) in 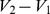 and is called the set of *additional compound nodes* (resp., *additional reaction nodes*).

Let 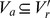 be a set of additional reaction nodes. In the Boolean model, each node is assigned either “0” or “1”. For a compound node, “1” means producible and “0” means not producible. As for a reaction node, “1” means active and “0” means inactive. Let 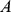 be such an assignment (that is 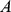 is a function from 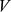 to 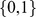). For each node 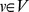, we write 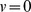 (resp., 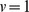) if 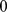 (resp., 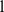) is assigned to 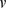. 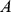 is called a *valid assignment* if the following conditions are satisfied: (i) for each 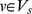, 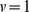. (ii) for each 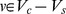, 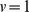 if and only if there is 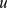 such that 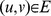 and 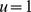. (iii) for each 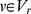, 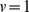 if and only if 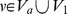 and 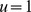 holds for all 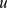 such that 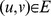. This implies that each reaction node corresponds to an “AND” node and each compound node corresponds to an “OR” node.

If 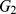 has no directed cycles, a valid assignment is uniquely determined for each 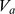. However, if 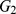 has a directed cycle, multiple valid assignments may exist. Let us call 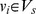 and 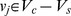 *source connected* if there is a directed path from 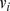 to 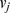, and the values of the nodes included in the path are all 1. There exist valid assignments where the values of nodes in a directed cycle are 1 even if these nodes are not source connected. In order to avoid such a case, we use the notion of *minimal valid assignment*, which is similar to the notion of maximal valid assignment defined in [32]. A valid assignment 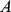 is called *minimal* if 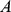 is valid and 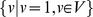 is minimal with respect to the inclusion relationships for sets.

Now we define the **Minimum Reaction Insertion** as follows:

- **Input:** A host metabolic network 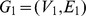, a reference metabolic network 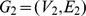, and a target compound 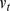.
- **Output:** A minimum cardinality set of 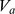 for which 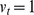 is satisfied in the minimal valid assignment of the induced subgraph of 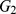 by 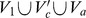.

As mentioned in the section of Theoretical Results, a minimal valid assignment is uniquely determined if 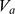 is given. However, solving MRI is not easy since the number of candidate 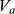 is 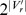 and MRI is proved to be NP-complete. Since utilizing software packages of Integer Programming (IP) is efficient for solving NP-complete problems, we develop a method of IP formalization for solving MRI. Since the computational time of the IP-based method is considered to be exponential in terms of the number of variables, it is important to develop an IP formalization of MRI with a small number of variables. To do so, our previously developed method for **Minimum Reaction Cut** (MRC) [32] may be useful although many modifications are necessary.

MRC is a problem to find a minimum set of reactions that interfere with the production of target compounds [32] and is known to be NP-complete. Let 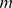 (resp., 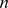) be the number of compound (resp., reaction) nodes. If we use 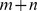 time steps to calculate the maximal valid assignment in MRC, the number of variables in IP is 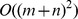. The feedback vertex set (FVS) is a node set whose removal makes a network cycle-free. In [32], we succeeded in reducing the number of variables to 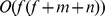, where 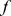 is the size of the feedback vertex set and 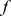 is considerably smaller than 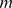 or 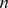. If use of 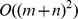 variables is allowed in MRI, almost the same method as in MRC can be used. However, to reduce the number of variables in IP to 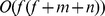, many modifications are necessary since minimal valid assignment and maximal valid assignment have different features.

### Integer Programming-Based Method for Minimum Reaction Insertion

Here, we show IP formalization methods for MRI in the Boolean model. To apply IP, problems must be formalized to maximize or minimize a given objective function which is a linear function of integer variables and constraints must also be given as linear equations or inequations of integer variables.

Suppose that the host network and the reference network are given as shown in Fig. 2. The simplest IP formalization **IP-MRI-A** for solving **Minimum Reaction Insertion** is as follows where the time step increases by 1 when the Boolean calculation is synchronously conducted for every node:

**IP-MRI-A**

**Minimize**

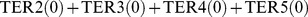
(1)


**Subject to**

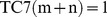
(2)


**for all** 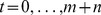


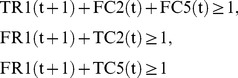
(3)




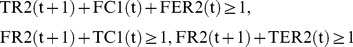
(4)




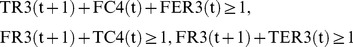
(5)




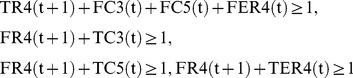
(6)




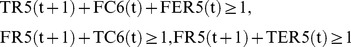
(7)




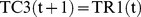
(8)




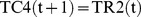
(9)




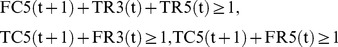
(10)




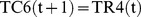
(11)




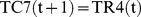
(12)




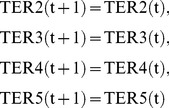
(13)




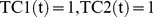
(14)




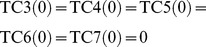
(15)




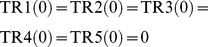
(16)




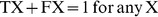
(17)where every variable takes either 0 or 1. 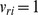 (resp., 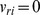) at time step 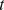 is represented by TR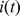 = 1 (resp. FR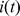 = 1) and TR
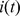
+FR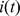 = 1 holds for any 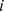 and 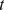. For example, TR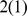 = 0 means that 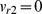 at time step 1, and FR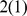 = 1 automatically holds at the same time. In the implementation, FR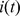 is replaced with 1-TR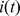 to reduce the number of variables. Similarly, the values of compound nodes are represented by TC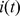 and FC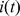. For example, FC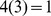 means that 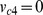 at time step 3.

(3) represents the Boolean relation 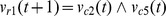. Since Boolean relations such as “
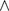
” or “
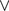
” cannot directly be used in IP, it is necessary to convert them into linear equations and/or inequations. Since 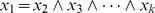 can be represented by 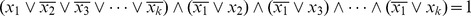, 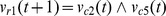 can be converted into 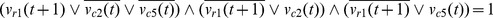, and then (3) is obtained.

For a compound node with indegree 1, the value of the predecessor node is just copied. For example, since 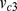 has only one predecessor 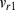, 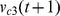 is just copied from 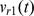 as shown in (8). Similarly, 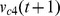 is just copied from 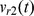 as shown in (9).

For a compound node with indegree more than 1, it is necessary to convert the “
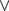
” relation into linear equations or equations. (10) represents the Boolean relation 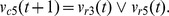
Since 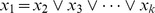 is represented by 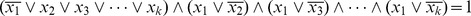, 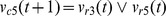 can be converted into 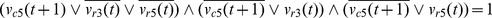, and then (10) is obtained.

As for the reaction nodes not included in the host network, TER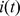 and FER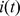 are used to represent whether 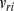 is activated. We use a virtual node 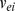 as one of the predecessors of 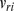. Since 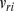 is represented by an AND node, 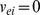 keeps 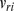 inactive even if all other predecessors of 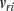 are 1. For example, 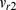 in Fig. 2 has only one predecessor 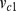. However, since 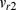 is not included in the host network and 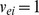 is necessary for 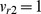, 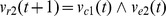 must hold, and then (4) is obtained.

Since we assume minimal valid assignment, at 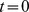, the source compound nodes are assigned 1, but the other compound nodes and reaction nodes are assigned 0.


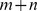
 is the largest number of time steps necessary for the 0–1 assignment to converge. (1) means that the number of additional reactions should be minimized. (2) means that the target compound 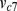 should become 1 after the 0–1 assignment converges. (3)–(7) represent the constraints by 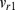 to 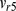 respectively. Note that 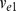 does not exist since 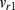 is included in the host network and then 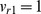 holds for any 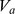. (8)–(12) represent the constraints by 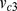 to 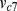 respectively. (13) represents that 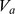 does not change by time transition. (14) means that 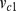 and 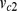 are source nodes. (15)–(16) represent that all nodes but source nodes are assigned 0 in the initial state. (17) means that “T” and “F” represent “true (1)” and “false (0)” respectively, and complement each other.

The above formalization can clearly solve MRI and obtain the correct solution 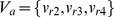, however 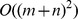 variables are necessary. To reduce the number of variables, it is necessary to reduce the number of time steps. If time is not taken into account at all, the following inappropriate IP formalization **IP-MRI-B** is obtained.

**IP-MRI-B**

**Minimize**

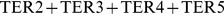
(18)


**Subject to**

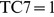
(19)




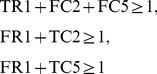
(20)




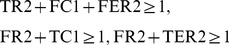
(21)




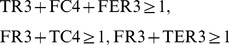
(22)




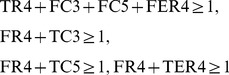
(23)




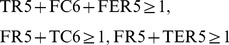
(24)




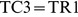
(25)




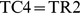
(26)




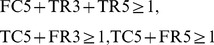
(27)




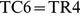
(28)




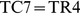
(29)




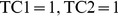
(30)




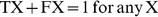
(31)


When compared to IP-MRI-A, (18),(19),(20)–(24), (25)–(29), (30), and (31) correspond to (1),(2),(3)–(7), (8)–(12), (14), and (17) respectively although the notion of time is not used in IP-MRI-B. (13) in IP-MRI-A means that the value of 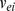 does not change in the time transition, but this constraint is not necessary for IP-MRI-B since it does not have the notion of time. Moreover, neither (15) nor (16) of IP-MRI-A is used in IP-MRI-B.

In IP-MRI-B, the solution of IP is 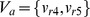 since 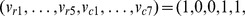 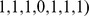 is a valid assignment and satisfies 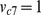. Note that 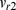 and 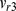 are forced to be 0 since they are not included in either the host network or 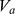. Although it satisfies all constraints and 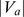 is minimum, this assignment is not appropriate since 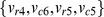 forms a cycle and all of them are assigned 1 without the influence of source nodes. To avoid such an inappropriate assignment, it is necessary to consider minimal valid assignment with respect to the number of 1 s for each 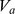. As shown in the section of Theoretical Results, the minimal valid assignment is uniquely determined for each 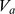.

Thus, IP-MRI-A can solve MRI, but 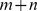 time steps are necessary, while IP-MRI-B, which does not use the notion of time, cannot solve MRI. The feedback vertex set (FVS) is a set of nodes whose removal makes the network acyclic. Since IP-MRI-B can solve MRI if there is no cycle, it is reasonable to apply IP-MRI-B for the acyclic network obtained by the deletion of FVS and use the notion of time as in IP-MRI-A to nodes included in 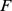 based on the idea developed in [32].

**Figure 3:**
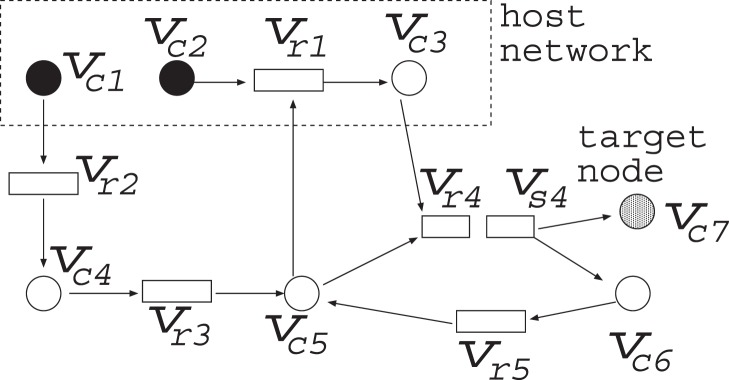
The cycles are decomposed in the FVS-based method.

In the improved method, IP-MRI-C, we firstly find an FVS 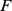 consisting of reaction nodes and then decompose each 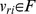 into two nodes 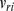 and 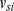 so that 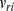 has only in-edges and 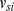 has only out-edges. For example, in the network of Fig. 2, since 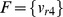 is a feedback vertex set, 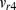 is decomposed into 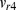 and 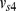 as shown in Fig. 3. Furthermore, we put an additional constraint that 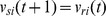. The number of time steps of IP-MRI-C is 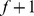 while that of IP-MRI-A is 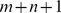, where 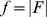. Therefore, the numbers of variables in IP-MRI-C and IP-MRI-A are 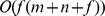 and 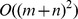 respectively. Since 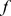 is considerably smaller than 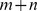 in most metabolic networks and the computational time of IP exponentially increases with the number of variables, we can expect a significant improvement from the view point of the computational time.

Although finding the minimum FVS is known to be NP-complete, it is not necessary to use the minimum FVS in our problem setting. We use a simple greedy algorithm to choose FVS as follows:

Procedure 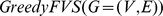, where 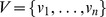 and 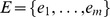

 
**for** 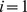 to 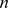 **do**
  

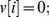

 
**for** 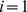 to 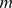 **do**
  

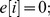

 

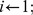

 
**while** there exists 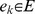 such that 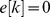

  

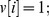

  
**if** there exists 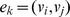 such that 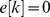 and 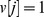 **then**
   

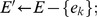

   

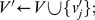

   
**for all** 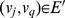 **do**
    

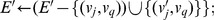

   

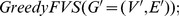

  
**else if** there exists 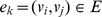 such that 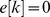 and 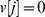 **do**
   

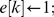

   

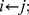

  
**else if** there exists 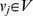 such that 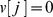 **do**
   

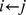
 for the minimum 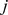;
**return** 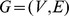;

Since the reaction nodes for FVS are chosen by a greedy algorithm, the size of FVS is not always optimal. However, it is important to note that even if the size of FVS is not optimal, the solution of MRI calculated by IP-MRI-C is always optimal. If there are multiple optimal solutions in MRI, there is a possibility that the solutions are different since IP outputs only one solution. However, it may be possible to enumerate all optimal solutions of MRI by iteratively solving IP with a constraint to avoid the already chosen solutions.

For example, IP-MRI-C for Fig. 2 is as follows, where 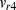 is decomposed into 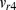 and 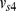, and time step increases by 1 only when the value of 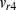 is copied to 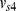.

**IP-MRI-C**

**Minimize**



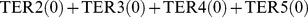
(32)


**Subject to**

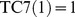
(33)


**for all** 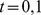


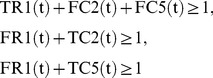
(34)




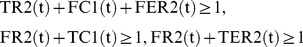
(35)




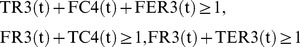
(36)




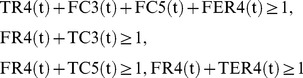
(37)




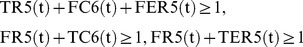
(38)




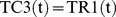
(39)




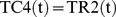
(40)




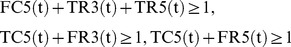
(41)




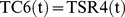
(42)




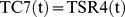
(43)




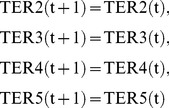
(44)




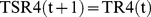
(45)




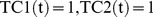
(46)




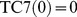
(47)




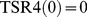
(48)




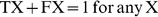
(49)where each variable takes either 0 or 1.

When compared to IP-MRI-A, (32),(33),(34)–(38), (39)–(43), (44), (46), and (49) correspond to (1),(2),(3)–(7), (8)–(12), (13), (14), and (17), respectively. 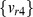 is chosen as a feedback vertex set, and then decomposed into 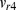 and 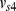 as shown in Fig. 3.

Note that the number of time steps is 2 = 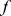
+1, and TSR4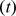 = 1 represents 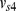 = 1. In (42)–(43), the constraints for 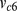 and 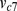 are represented by the variable corresponding to 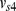 instead of that to 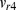. (45) represents that the time step increases by 1 when the value of 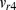 is copied to 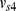 in Fig. 3. (48) represents 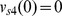 to obtain the minimal valid assignment.

**Figure 4:**
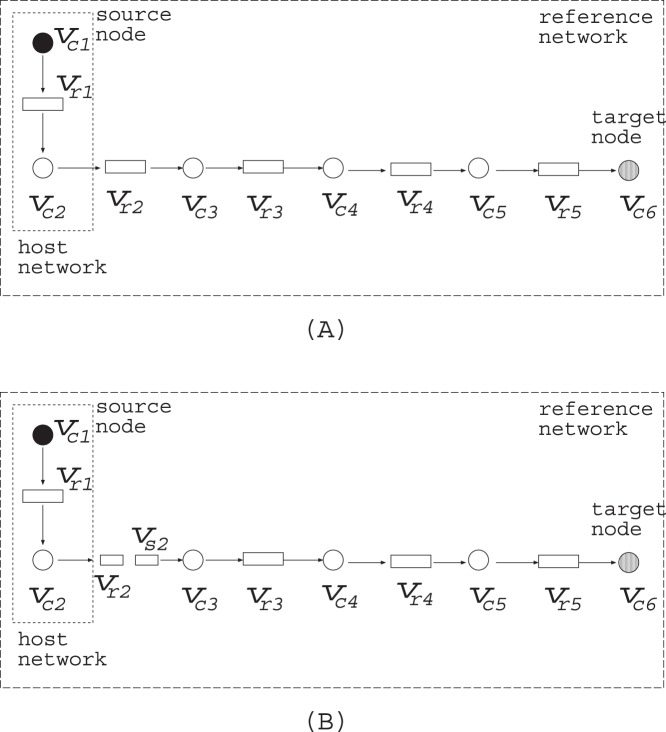
A special case where there is no cycle. (A) An example where a contradiction occurs if the notion of time is not used. (B) Decomposition of a border node to avoid the contradiction.

Additionally, if we use the FVS-based method and no cycles are included in 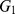 and 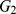, the number of necessary time steps is only one. For example, suppose that 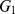 and 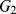 are as shown in Fig. 4 (A). In this case, the correct solution of MRI is 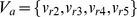. However, if we set TC1(0) = 1 and TC6(0) = 0, IP can output no solution since the condition TC6(0) = 1 is never satisfied. On the other hand, if we set TC1(0) = 1 and TC6(0) = 1, an inappropriate solution 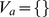 is obtained by IP. To avoid such a case, in our method, if one of the predecessors of an additional reaction node 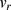 is included in the host network, we decompose 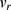 as if it were included in FVS. For example, in the network of Fig. 4 (A), 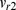 is decomposed into 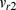 and 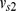 as shown in Fig. 4 (B) so that the values of the source nodes and the target node are calculated in different time steps.

## Results

### Computer Experiments

We conducted computer experiments for solving MRI with data downloaded from the KEGG database. The experiment was conducted on a PC with an Intel(R) Xeon(R) 3.33 GHz CPU and 10 GB RAM having the SUSE Linux (version 12.2) operating system, where CPLEX (version 12.4.0.0) was used as the solver of integer programming.

In this study, a reference network consists of the central metabolism and the related modules necessary for producing the target compound. A map of the KEGG PATHWAY is a minimum unit, and three or four maps of the KEGG PATHWAY are chosen and integrated as the reference network in each of our experiments. As for species, a reference network includes the chemical reactions of all species, whereas the metabolic networks of *E. coli* are used for the host networks. The major difference between the pathway alignment methods by KEGG and our developed method is that our method is based on a Boolean model, whereas the pathway alignment methods consider only the topology of networks.

In synthetic biology, it is of great interest to construct a minimal genome that realizes the desired functions [33]–[35]. Since glycolysis, gluconeogenesis, citrate cycle and pentose phosphate pathway are considered to be essential even in artificial organisms, it is reasonable to assume that the host networks in the computer experiments have some of these pathways in one of the simplest organisms, *E. coli*. Because the purpose of this study is not focused on the reconstruction of genome-scale metabolic network model, but the design of a minimal genome in addition to the existing pathways to produce a desired compound, each reference network consists of the maps of the KEGG pathway located between the central metabolism and each target compound.

**Figure 5:**
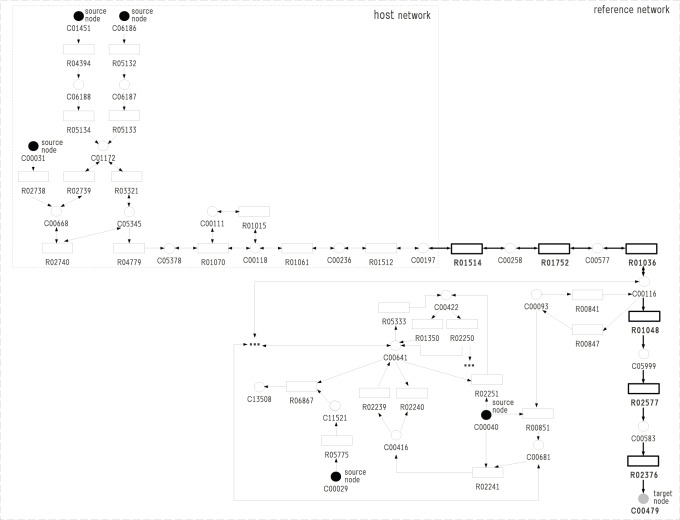
Propanol (C00479) becomes producible from glycolysis and gluconeogenesis by the addition of *V_a_* = {R01514, R01752, R01036, R01048, R02577, R02376}.

In the first computer experiment, the target compound is propanol (C00479 in KEGG ID), the host network is glycolysis and gluconeogenesis of *E. coli* (eco00010.xml), and the reference network covers glycolysis, gluconeogenesis and glycerolipid metabolism of other species (ko00010.xml and ko00561.xml). The numbers of compound and reaction nodes are 58 and 85, respectively, where 30 reactions are reversible. The source nodes are D-glucose (C00031), oxaloacetate (C00036), salicin (C01451), arbutin (C06186), UDP-glucose (C00029), acyl-CoA (C00040), and diglucosyl-diacylglycerol (C06040), which are represented by black circles in Fig. 5. It took 0.19 s to solve MRI. The obtained additional reactions are 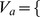
R01514, R01752, R01036, R01048, R02577, R02376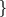, where these reactions produce propanol from 3-phospho-D-glycerate (C00197) via glycerol (C00116) as shown in Fig. 5. Since 3-phospho-D-glycerate (C00197) is producible by glycolysis and gluconeogenesis of *E. coli* and works as a connection between glycolysis and glycerolipid metabolism, the obtained 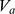 can be considered an appropriate solution of MRI.

### Difference between Developed Model and Shortest Path-Based Model

**Figure 6:**
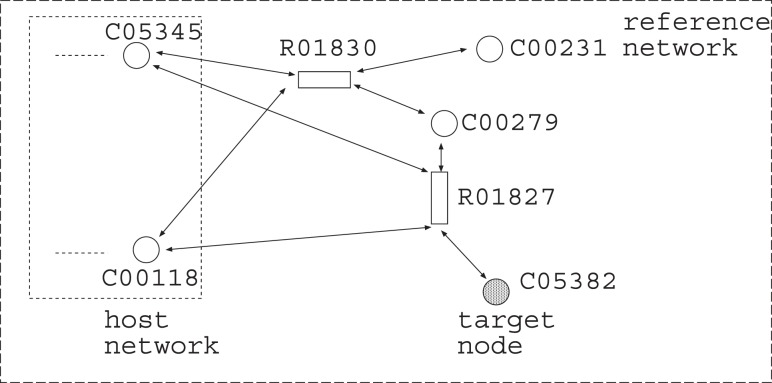
When the target compound was C05382, MRI selected R01827 and R01830 from 66 candidates for the additional reactions whereas the shortest path-based method (PathComp) selected only R01827.

To show the difference between the developed model and the shortest path-based models, we conducted the second experiment where PathComp of KEGG (“http://www.genome.jp/tools/pathcomp/”) was used to calculate the solution of the shortest path-based model. In the experiment, the host network consists of glycolysis, gluconeogenesis and citrate cycle of *E. coli* (eco00010.xml and eco00020.xml), and the reference network consists of glycolysis, gluconeogenesis, citrate cycle and pentose phosphate pathway of other species (ko00010.xml, ko00020.xml and ko00030.xml). The numbers of compound and reaction nodes are 64 and 108, respectively, where 59 reactions are reversible. There are four source nodes, D-glucose(C00031), arbutin(C06186), salicin(C01451), and acetate (C00033), and the number of candidates for the additional reactions is 66. When the target compound is sedoheptulose 7-phosphate (C05382), as shown in Fig. 6, the solution of MRI is 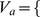
R01827, R01830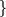, where the substrates of R01827 are beta-D-fructose 6-phosphate (C05345) and D-erythrose 4-phosphate (C00279). It took 32.58 s to obtain the solution. Since D-erythrose 4-phosphate (C00279) is not included in the host network, it is necessary to add R01830 in which substrates are beta-D-fructose 6-phosphate (C05345) and D-glyceraldehyde 3-phosphate (C00118) and the products are D-xylulose 5-phosphate (C00231) and D-erythrose 4-phosphate (C00279). It is to be noted that both beta-D-fructose 6-phosphate (C05345) and D-glyceraldehyde 3-phosphate (C00118) are producible by the host network.

On the other hand, PathComp just connects the producible compounds and the target compound adds only R01827 since R01827 is adjacent to both beta-D-fructose 6-phosphate (C05345) and sedoheptulose 7-phosphate (C05382). However, it is clear that R01827 does not occur if D-erythrose 4-phosphate (C00279) does not exist. Thus the difference between the shortest path-based method and the developed method is that the developed method considers Boolean constraints for each reaction and compound whereas the shortest path-based method only considers the connectivity of nodes.

### Scalability

Next, we conducted the third experiment to show the scalability of our method. The host network consists of the source nodes of glycolysis and gluconeogenesis of *E. coli* (eco00010.xml), that is, D-glucose(C00031), arbutin(C06186), salicin(C01451), oxaloacetate(C00036) and acetate (C00033). The reference network consists of glycolysis, gluconeogenesis, citrate cycle, pentose phosphate pathway and butanol metabolism of other species (ko00010.xml, ko00020.xml, ko00030.xml and ko00650.xml), where R01172 is treated as a reversible reaction. The target compound is butanol (C06142). The numbers of compound and reaction nodes are 93 and 150, respectively, where 87 reactions are reversible. It took 919.79 s (15m19s) for the developed method to solve MRI and the solution was 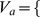
R00235, R00238, R01977, R03027, R01171, R01172, R03545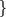. These seven reactions form a path from acetate to 1-butanol via acetyl-CoA, acetoacetyl-CoA, crotonoyl-CoA and butanoyl-CoA, which satisfies the Boolean constraints. Since the number of reactions in the reference network is 150, it is necessary to examine 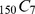 cases if an exhaustive search is conducted. Since examining 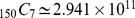 cases is almost impossible, it is seen that the IP-based method is useful for solving MRI, particularly when the given networks are not small.

### Difference between Developed Model and FBA-Based Model

**Figure 7:**
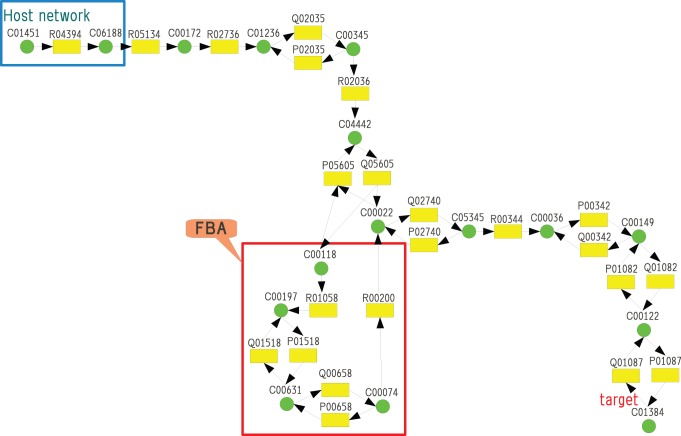
The comparison between the Boolean model and the FBA-based model [20]. More reactions are necessary for the FBA-based model to produce the target compound than the Boolean-based model.

Finally, we conducted an experiment to show the difference between the developed model and the FBA-based model. We assume that the reference network consists of glycolysis, gluconeogenesis, citrate cycle, pentose phosphate pathway and butanol metabolism of other species (ko00010.xml, ko00020.xml, ko00030.xml and ko00650.xml), and the host network includes only one reaction R04394 between salicin (C01451) and salicin 6-phosphate (C06188) as shown in Fig. 7. Therefore, the source node is only salicin (C01451). Note that reversible reactions are decomposed into two reactions, and denoted by P and Q. The target compound is maleic acid (C01384). The numbers of compound and reaction nodes are 93 and 150, respectively, where 87 reactions are reversible.

Then, the solution of MRI in our Boolean model is {R05134, R02736, R02035, R02036, R05605, R00344, R00342, R01082, R01087}, whereas the solution of FBA-based model is {R05134, R02736, R02035, R02036, R05605, R01058, R01518, R00658, R00200, R00344, R00342, R01082, R01087}. It is to be noted that {R01058, R01518, R00658, R00200} is not necessary for the Boolean model, but necessary for the FBA-based model. In the Boolean model, R01058 is not necessary to produce C01384 since the lack of reactions in downstream does not affect. However, in the FBA model, R01058 is necessary. Otherwise, C00118 is not consumed and then R05605 (denoted as Q05605 in Fig. 7) cannot occur. Thus, the solution of MRI in the FBA-based model tends to include more reactions than that in the Boolean model. It took 7896.46 s (2h11m36s) to solve the Boolean model of MRI.

### Theoretical Results

Although solving IP is NP-complete, a problem that can be formalized as IP is not always NP-complete. Therefore, in the following paragraphs, we prove that MRI is NP-complete and show the appropriateness of formalizing MRI of the Boolean model as IP.

**Theorem 1:** **Minimum Reaction Insertion** is NP-complete even when the maximum indegree and outdegree are bounded by 2.

**Figure 8:**
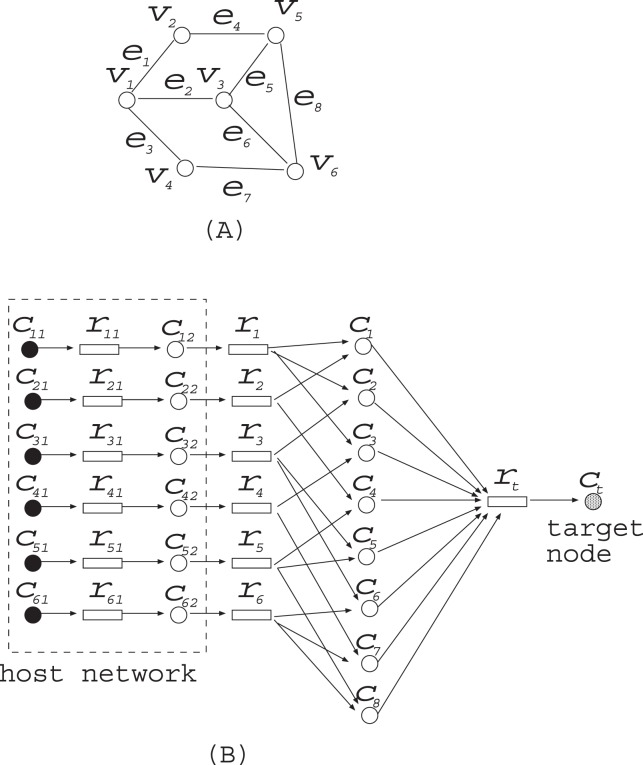
The polynomial time reduction from minimum vertex cover (MVC) problem to minimum reaction insertion (MRI) problem. (A) An instance of MVC. (B) The corresponding instance of MRI.

*Proof:* Since the problem is clearly in NP, it suffices to show NP-hardness. The proof is by a polynomial time reduction from minimum vertex cover (MVC), which is a problem for a given graph to find the minimum number of nodes so that each edge is incident to at least one of the selected nodes. For example, for the graph shown in Fig. 8(A) , 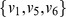 is an optimal solution of MVC.

Let 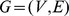 be an instance of MVC, where 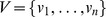 and 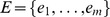. We construct the corresponding MRI as follows. The host network 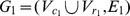 is given by
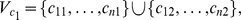


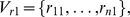


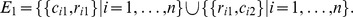



The reference network 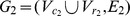 is given by
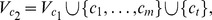


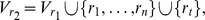


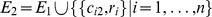


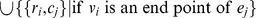


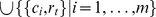


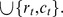



For example, MVC for the graph shown in Fig. 8 (A) is converted into MRI shown in Fig. 8 (B). It is clear that this conversion can be done in polynomial time.

**Figure 9:**
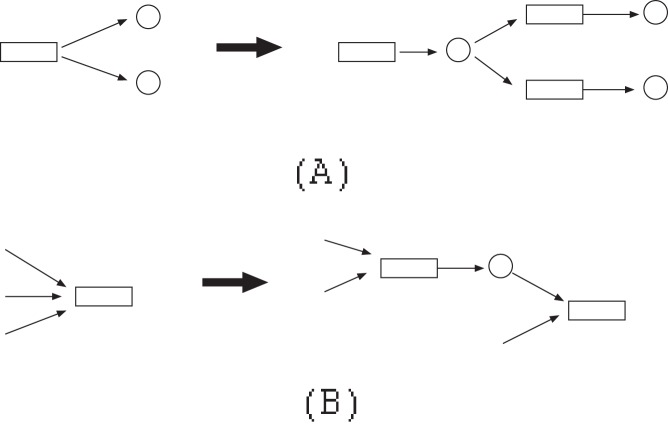
The conversion of nodes with the indegree and the outdegree more than 2.

In the following paragraphs, we show that MVC for 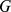 has a solution of size 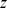 if and only if MRI has a solution in which 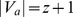 holds. When 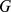 has a vertex cover of size 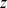, 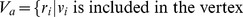 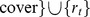 satisfies 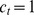 in the minimal valid assignment and 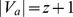 holds. On the other hand, suppose that 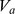 satisfies 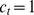 in the minimal valid assignment and 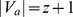. Since 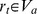 is necessary for 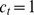, 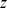 nodes are included in 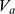 from 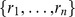. Since each 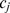 must be 1 to satisfy 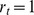, at least one predecessor of each 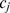 must be included in 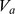 for each 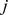. Since there is an edge between 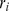 and 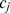 if and only if 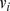 is incident to 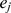, 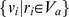 is a vertex cover of size 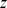. Nodes whose degrees are more than 2 can be converted by the methods shown in Fig. 9.

**Theorem 2:** Given a host network, a reference network and a set of additional reactions, a minimal valid assignment is uniquely determined.

*Proof:* For any valid assignment 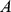, the assignment obtained by assigning 0 to all nodes that are not source connected is also a valid assignment. On the other hand, for any valid assignment 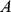, the assignment obtained by assigning 0 to a source connected node is not a valid assignment. Since source connected nodes 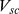 are uniquely determined for 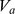, 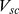 is a minimal valid assignment and uniquely determined.

## Discussion

In this paper, we formalized an optimization problem MRI in a Boolean model with a notion of minimal valid assignment. We proved that MRI in the Boolean model is NP-complete and the minimal valid assignment is uniquely determined when 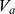 is given. Since an exhaustive search cannot be used to solve MRI when the given networks are not small, we developed an IP-based method for MRI. To improve the scalability of the developed method, it is necessary to reduce the number of variables appearing in IP formalization since the computational time of IP is considered to be exponential to the number of variables. Although the simple IP formalization with the notion of time is useful for solving MRI, it needs 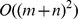 variables in IP formalization. If the notion of FVS is used, the number of necessary time steps reduces to 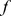, where 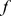 denotes the size of FVS, and the number of variables in IP is 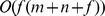. Although the idea of using FVS is similar to [32], many modifications are necessary since the minimal valid assignment and the maximal valid assignment have many different properties.

We also conducted four computer experiments in which data were downloaded from the KEGG database, CPLEX was used as the IP solver, and propanol, butanol, sedoheptulose 7-phosphate, and maleic acid were used as the target compound for each experiment. The host network was a metabolic network of *E. coli* and the reference network of KEGG was used as the reference network. The results of the computer experiments confirmed the correctness and the scalability of the developed method, and the appropriateness of the problem setting of MRI.

An important advantage of our Boolean model is its capability of detecting the lack of substrates, whereas the connectivity-based methods cannot appropriately handle this point. An extended type of connectivity-based method is BNICE, which enumerates all possible pathways from the source nodes to the target compound, and uses thermodynamical feasibility and pathway length to evaluate each candidate pathway. In contrast, the developed method evaluates each candidate pathway based on the number of additional reactions. Another advantage of the developed model is its capability of handling branches and/or cycles in a pathway from the source compounds to the target compound, whereas BNICE considers only the non-branching paths. However, since BNICE nicely evaluates each pathway by the thermodynamic free energy of the included compounds and length, considering the thermodynamic free energy in a Boolean model represents an important direction of our future work.

It is to be noted that the solution of MRI in the FBA-based model is different from that in the Boolean model. In particular, if the reference network includes a large redundant part, the FBA-based model tends to output a larger solution than the Boolean model, although the FBA-based model is very fast when compared to the Boolean model. Therefore, one of our future works is to develop a hybrid method combining the FBA-based method and the Boolean-based method. Petri-net-based methods [36] are also interesting since they may extract the good points of both Boolean-based methods and FBA-based methods.
